# Impact of repeated annual community directed treatment with ivermectin on loiasis parasitological indicators in Cameroon: Implications for onchocerciasis and lymphatic filariasis elimination in areas co-endemic with *Loa loa* in Africa

**DOI:** 10.1371/journal.pntd.0006750

**Published:** 2018-09-18

**Authors:** Samuel Wanji, Winston Patrick Chounna Ndongmo, Fanny Fri Fombad, Jonas Arnaud Kengne-Ouafo, Abdel Jelil Njouendou, Yolande Flore Longang Tchounkeu, Benjamin Koudou, Moses Bockarie, Grace Fobi, Jean Baptiste Roungou, Peter A. Enyong

**Affiliations:** 1 Parasites and Vector Biology research unit (PAVBRU), Department of Microbiology and Parasitology, University of Buea, Buea, Cameroon; 2 Research Foundation for Tropical Diseases and the Environment (REFOTDE), Buea, Cameroon; 3 Centre for Neglected Tropical Diseases (incorporating the Lymphatic Filariasis Support Centre), Liverpool School of Tropical Medicine, Liverpool, United Kingdom; 4 African Program for Onchocerciasis Control (APOC), Ouagadougou, Burkina Faso; Task Force for Child Survival and Developmentorce for Global Health, UNITED STATES

## Abstract

**Background:**

Loiasis is a filarial infection endemic in the rainforest zone of west and central Africa particularly in Cameroon, Gabon, Republic of Congo, and Democratic Republic of the Congo. Repeated treatments with ivermectin have been delivered using the annual community directed treatment with ivermectin (CDTI) approach for several years to control onchocerciasis in some *Loa loa-Onchocerca volvulus* co-endemic areas. The impact of CDTI on loiasis parasitological indicators is not known. We, therefore, designed this cross sectional study to explore the effects of several rounds of CDTI on parasitological indicators of loiasis.

**Methodology/Principal findings:**

The study was conducted in the East, Northwest and Southwest 2 CDTI projects of Cameroon. Individuals who consented to participate were interviewed for ivermectin treatment history and enrolled for parasitological screening using thick smears. Ivermectin treatment history was correlated with loiasis prevalence/intensity. A total of 3,684 individuals were recruited from 36 communities of the 3 CDTI projects and 900 individuals from 9 villages in a non-CDTI district. In the East, loiasis prevalence was 29.3% (range = 24.2%–34.6%) in the non-CDTI district but 16.0% (3.3%–26.6%) in the CDTI district with 10 ivermectin rounds (there were no baseline data for the latter). In the Northwest and Southwest 2 districts, reductions from 30.5% to 17.9% (after 9 ivermectin rounds) but from 8.1% to 7.8% (not significantly different after 14 rounds) were registered post CDTI, respectively. Similar trends in infection intensity were observed in all sites. There was a negative relationship between adherence to ivermectin treatment and prevalence/intensity of infection in all sites. None of the children (aged 10–14 years) examined in the East CDTI project harboured high (8,000–30,000 mf/ml) or very high (>30,000 mf/ml) microfilarial loads. Individuals who had taken >5 ivermectin treatments were 2.1 times more likely to present with no microfilaraemia than those with less treatments.

**Conclusion:**

In areas where onchocerciasis and loiasis are co-endemic, CDTI reduces the number of, and microfilaraemia in *L*. *loa*-infected individuals, and this, in turn, will help to prevent non-neurological and neurological complications post-ivermectin treatment among CDTI adherents.

## Author summary

*Loa loa* (the parasite causing loiasis), also known as African eye worm, is endemic in forest areas of west and central Africa. In several of the endemic areas, it co-exists with onchocerciasis and lymphatic filariasis (LF). Because of the benefit individuals suffering from onchocerciasis could have by taking ivermectin where the disease is severe, despite the risk of developing serious side-effects due to being co-infected with *L*. *loa*, mass drug administration (MDA) of ivermectin for the control of onchocerciasis has been ongoing in areas where the two diseases overlap. Ivermectin is also effective against loiasis. It is, therefore, hypothesized that several years of ivermectin MDA against onchocerciasis in those areas may have impacted on parasitological indicators for loiasis. In particular, we assess the impact of annual community directed treatment with ivermectin (CDTI) on loiasis with specific reference to the relationship between adherence to treatment and the risk of developing severe (nervous system) complications following ivermectin treatment. We also discuss the feasibility of eliminating onchocerciasis and/or LF in areas endemic for *L*. *loa* with ivermectin as the sole intervention tool.

## Introduction

Loiasis is a filarial infection endemic in the rainforest areas of West and Central Africa, particularly in Cameroon, Gabon, Republic of Congo and Democratic Republic of the Congo (DRC) [1, 2], where its distribution is associated with that of its vectors *Chrysops silacea* and *C*. *dimidiata*. The common clinical signs of loiasis are the subconjunctival migration of the adult worm (reported for the first time by Mongin [3], Calabar swellings, pruritus, oedema and arthralgia. Loiasis can also be rarely associated with renal, cardiac, neurologic or chorioretinal complications ([4, 5]. In some regions it has been noted that loiasis is the second or third most common reason of medical consultation, after malaria and pulmonary diseases [6]. Chesnais and colleagues revealed that high-grade *L loa* microfilaraemia was associated with an increased mortality risk, further suggesting that loiasis is not a benign condition and merits more attention because of its effect on onchocerciasis and Lymphatic Filariasis (LF) control strategies [7].

It has been demonstrated that a single oral dose of ivermectin brings about a dramatic decrease in the load of *Loa loa* microfilariae (mf) and even an improvement in some clinical signs related to the infection [8–15]. The impact of repeated large-scale 3-monthly treatment with ivermectin for over a year on the transmission of loiasis in a forest village in south Cameroon where loiasis was highly endemic, with a prevalence above 30% was investigated by Chippaux and colleagues [16]. The prevalence of loiasis was reduced to less than 10% and the mean microfilaraemia was decreased by 90% over a 2-year period. The infection rate (all stages) in *Chrysops* was decreased by 75% and the infective rate (percentage of *Chrysops* harbouring third-stage larvae of *L*. *loa* in the head) was decreased by 85% in *C*. *dimidiata* and became zero in *C*. *silacea*. This pilot study demonstrated that large-scale treatment with ivermectin could be a potential effective method for the control of *L*. *loa*.

Since the early 1990s, it was observed that ivermectin, the drug of choice for onchocerciasis and LF control in Africa, can induce Severe Adverse Events (SAEs) in individuals harbouring high densities of *Loa* mf in the blood [13, 17]. The condition consists of a potentially fatal encephalopathy occurring within 2–3 days after treatment [18, 19]. In non-fatal cases, long-term neurologic sequelae may occur. Since 1991, more than 400 cases of such neurologic SAEs have been reported from Central Africa, mainly from Cameroon and DRC. For this reason, the use of ivermectin on a large-scale basis for the control of LF in loiasis-endemic areas has not been promoted and it has been considered unethical for many areas where the disease is endemic. However, since ivermectin has a direct beneficial effect on the clinical manifestations of onchocerciasis, and these benefits outweigh the risk of central nervous system adverse events (particularly in highly onchocerciasis endemic areas), mass ivermectin distribution for the control of *Onchocerca volvulus* has been approved in *Loa* endemic areas where onchocerciasis co-occurs and where the prevalence of *Onchocerca* nodules exceeds 20% in the adult population (indicating meso- to hyperendemicity). This is done provided that an efficient surveillance system is put in place to promptly identify and manage SAE cases [20].

A significant proportion of Community Directed Treatment with Ivermectin (CDTI) projects of the African Programme for Onchocerciasis Control (APOC) is situated in areas where onchocerciasis and loiasis are co-endemic. This is the case in countries such as Nigeria, Cameroon, DRC and Angola. In Cameroon, amongst the 15 CDTI projects, 13 are partially or entirely situated in areas of onchocerciasis-loiasis co-endemicity. For those Cameroon CDTI projects situated in areas of co-endemicity, eight of them have been distributing ivermectin for more than 10 rounds (11–13) and 5 of them for less than 10 rounds (6–8). Also important is the fact that some of the CDTI projects situated in areas of co-endemicity have historical data and even more recent data on the endemicity of loiasis before the introduction of CDTI. We, therefore, hypothesized that long-term ivermectin treatment areas of onchocerciasis-loiasis co-endemicity might have led to marked reductions in the clinical and parasitological indices of loiasis, together with significant reductions in the risk of developing SAEs in treated communities. This would surely constitute an added value to the primary goal of APOC, which was the control of onchocerciasis. This study was, therefore, designed to assess in selected populations: (i) the changes in the parasitological indices of loiasis (infection prevalence and intensity) that might have occurred under Mass Drug Administration (MDA) with ivermectin; (ii) the relationship between adherence to treatment and the prevalence/intensity of *L*. *loa* microfilaraemia; (iii) the impact of ivermectin MDA on the reduction of potential risk of developing SAEs; (iv) the relationship between adherence to treatment and the reduction in potential risk of developing SAEs. As a fifth objective, we also sought to evaluate *L*. *loa* infection in children in the East and Northwest CDTI projects of Cameroon, with the ultimate goal of estimating the protective effect of ivermectin MDA in preventing high microfilarial loads, hence minimising the risk of SAEs in children.

## Methods

### Study design and sampling strategy

**Fig 1:**
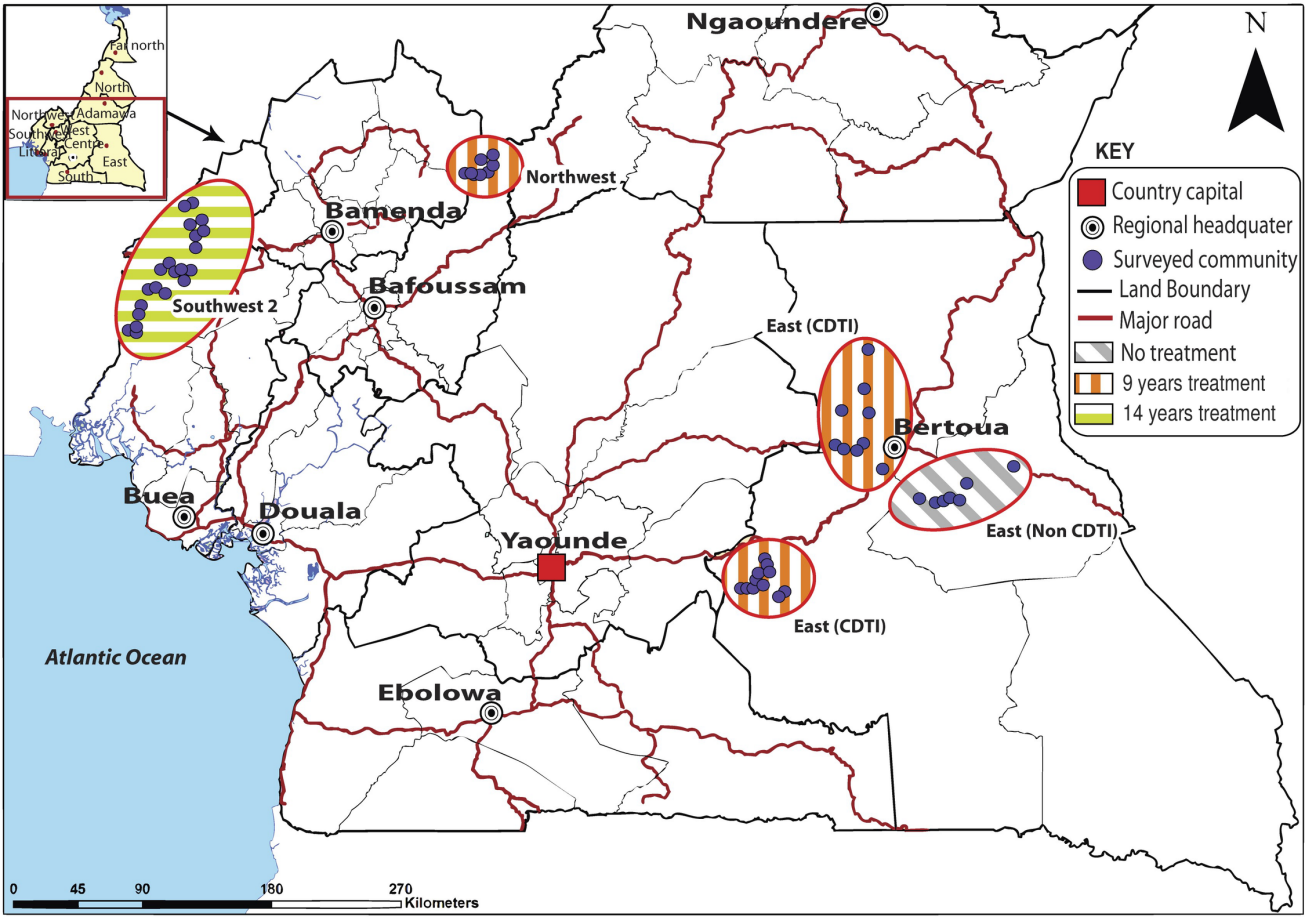
Map showing the locations of the study sites (QGIS software version 2.0.1).

This cross-sectional study was designed to evaluate the impact of CDTI on *L*. *loa* endemicity in four areas of co-endemicity with onchocerciasis in Cameroon (Fig 1). Three CDTI project sites were selected based on pre-control information on loiasis endemicity and these were the East, Northwest and Southwest 2 projects [21, 22]. However, there were no baseline data available for the East CDTI project. Therefore, a non-CDTI health district, also from the East and adjacent to one of the East CDTI sites, was selected. Although no formal comparison is truly possible between the CDTI and the non-CDTI areas in the East, inclusion of the latter provides useful information of the loiasis endemicity situation in an area of geographical proximity (Fig 1). A total of 36 and 9 geo-referenced communities were selected, respectively, from the three CDTI project sites and the non-CDTI health district. In each surveyed community, at least 80 adults (aged ≥15 years) and 20 children (aged 10-14years) of both sexes were screened.

In each community, ivermectin mass treatment was documented at the individual level and this information was obtained from oral declarations of participants following an interview on the number of times they had received ivermectin. We relied on oral information due to inconsistency on the availability of treatment registers in study communities [23]. Each individual who consented to participate (see below) was tested for blood mf using a thick blood film. The following parasitological indicators of *L*. *loa* were generated: mf prevalence, mf intensity, prevalence of low to moderate mf loads (1–8,000 mf/ml), prevalence of high mf loads (8,001–30,000 mf/ml) and prevalence of very high mf loads (>30,000 mf/ml). Indicators generated within CDTI projects were compared to pre-control indicators when baseline data were available (Northwest and Southwest 2 projects). Data on adults only were used in these comparisons. To further evaluate the impact of mass ivermectin treatment on loiasis, an association was investigated between adherence to the drug and the prevalence and intensity of mf infection. Adherence in this study was assessed using oral declaration and expressed as the number of times an individual had taken treatment since the inception of CDTI in his/her community. The reduction of potential SAE risk was estimated by comparing the proportion of individuals with high and very high *L*. *loa* mf loads (as defined above) before and under CDTI in the different study sites. The relationship between the adherence profiles to ivermectin treatment of community members and their risk of developing SAEs as anticipated by their *L*. *loa* microfilarial load was explored. SAEs were described as either individuals at risk of neurologic (in individuals harbouring >30000mf/ml of blood) or non neurologic complications (in individuals harbouring mf loads between 8001-30000mf/ml of blood) [19, 24].

### Study sites

The study was carried out in the East, Northwest and Southwest 2 CDTI project sites of Cameroon between March and October 2014 (Fig 1). The Southwest 2 CDTI project, situated in an area of *L*. *loa* mild endemicity, had started in the year 2000 and by the time of the study had been under CDTI for 14 years [25]. The East and Northwest CDTI projects, situated in areas of high *L*. *loa* endemicity, started much later (in 2004 and 2005 respectively) and so had, respectively, been under CDTI for 10 and 9 years prior to the study [21, 22, 26]. The East CDTI project consists of two sites, with one of them around the locality of Bertoua, the capital of the East Region of Cameroon (Fig 1). A fourth site, the CDTI-naïve health district in the East region was also surveyed.

The climate in the southwest and northwest is tropical with two seasons, one wet season of about 9 months, lasting from March to November and a short dry season from mid-November to mid-March. The mean annual rainfall in these areas varies from 2,500 to 4,000 mm. Ambient temperature ranges from 20°C to 40°C depending on the seasons. The climate of the east region is a Type A wet equatorial climate [27], with average temperatures of about 24°C with four seasons (a long dry season from December to May, a light wet season from May to June, a short dry season from July to October, and a heavy wet season from October to November). Humidity and cloud cover are relatively high, and precipitation averages 1,500–2,000 mm per year except in the extreme eastern and northern parts of the region, where it is slightly less.

### Eligibility criteria

Eligible individuals were individuals of both sexes that must have resided in the communities for at least 5 years and who accepted to participate in the study by signing the consent or assent forms provided by the research team.

### Ethical considerations

Prior to recruitment, the nature, objectives, benefits and potential risks of the study were explained to potential participants and those who agreed to take part in the study signed a consent form. Since some study participants were minors, their parents/guardians provided informed consent on their behalf. Recruitment was done based on the approach described previously by Kengne-Ouafo *et al*.[28]. The study protocol received ethical approval from the Cameroon National Ethics Committee, and the Research Ethics Review Committee of the Regional Office for Africa of the World Health Organization (WHO/AFRO).

### Assessment of adherence to treatment

Questions were asked as to whether individuals had ever taken ivermectin, the number of times and the last time he/she had taken the drug. Comparison of the records of the oral declarations with the records of the treatment registers of the Community Drug Distributors (CDDs) was not done because the latter were incomplete and, therefore, considered unreliable. Eligible participants were categorised by the number of times they had taken the drug ([0], [1–2], [3–4], [5–6], [7–8], [9–10], [11+] times), and underwent a parasitological (thick blood smear) examination. The correlation between the parasitological results thus obtained and the participants’ treatment profiles was investigated. Only participants who had not taken any filaricide medication for the past one year were enrolled in the study to avoid the situation in which a reduction in mf load could be due to the effect of a recently administered drug. Recruitment of the study participants was, therefore, carried out before the routine large-scale annual distribution of ivermectin in the villages of the CDTI projects.

### Parasitological methods

#### Blood collection and processing

Consenting individuals underwent parasitological examination as follows: a thick blood film was prepared from a standardized 50 μl finger prick blood collected under aseptic conditions between 10 am and 4 pm (given the diurnal periodicity of the human strain of *L*. *loa*) using a 75 μl non-heparinised capillary tube. A smear was prepared by spreading the blood on a clean and dry slide on an area of 1.5 x 2.5 cm. In the laboratory, the blood smears were dehaemoglobinised by immersing in tap water for 5–15 minutes and were then fixed with methanol for 1 minute. Smears were later stained in 10% giemsa for 45 minutes and allowed to dry [29].

#### Parasite identification

Parasites were identified using microfilarial identification keys [30]. Slides were read by trained technicians under a microscope at x10 magnification. The counts were expressed as microfilariae per millilitre (mf/ml) of blood. In these study areas, *Mansonella perstans*, another filarial species with blood-dwelling mf is co-endemic with *L*. *loa* [31], requiring the expertise of skilled technicians to identify *L*. *loa* in a stained film.

### Statistical analysis

Data were entered in a template created in EPI Info version 3.5.3 and analyzed with the Statistical Package for the Social Sciences (SPSS 20.0.0).

### Treatment history indicators

Examined individuals were grouped as either males or females, children (10–14 years of age) or adults (15+ years of age) and classified according to the number of times they had taken ivermectin. The different treatment classes were: [0], [1–2], [3–4], [5–6], [7–8], [9–10] and [11+].

### Parasitology indicators

The microfilarial prevalence was computed as the proportion of individuals, among those examined, harbouring mf in the blood (after microscopy). Microfilarial intensity was calculated as the arithmetic mean number of mf per ml of blood among those examined (i.e. including mf-positive and mf-negative individuals). Prevalence categories of mf load were calculated as: i) the proportion of individuals with low to moderate microfilaraemia (those with 1–8,000 mf/ml among examined); ii) the proportion of those with high mf load (individuals harbouring 8,001–30,000 mf/ml); and the proportion of individuals with very high mf loads (individuals harbouring > 30,000 mf/ml) as described in the Rapid Assessment Procedure for Loiasis (RAPLOA) report of 2001 [32].

Box-and-whiskers plots were used to summarize the overall mf prevalence and the prevalence of categories of mf load among the different CDTI projects according to the number of years under CDTI. This type of plot, enables visual inspection of the distribution of the data, with the box spanning the interquartile range, the horizontal line inside the box representing the median, and the whiskers extending to the lowest (minimum) and highest (maximum) observations.

Based on the fact that prevalence data are binomially distributed, the (baseline and post-CDTI) study periods were independent of each other, and the participants at the two study periods were different (not a longitudinal cohort but a cross-sectional study), differences between baseline and post-CDTI data in the proportions of mf positives, or of positives for each prevalence of mf load category, were analysed by the 2-sample z-test to compare sample proportions when this was an appropriate test (i.e. z times p-values >5) or a chi-square test applied to a 2×2 contingency table (without continuity correction as sample sizes were large) when the normal approximation of the binomial distribution could not be applied (i.e. sample size times the proportion (or 1 minus the proportion) < 5). The exact (Clopper-Pearson) 95% confidence intervals (95% CI) accompanying the calculated prevalence values are also provided.

The (non-parametric) Mann-Whitney U test was used to compare average loiasis infection intensities given that mf loads are typically over dispersed and therefore do not follow a normal (Gaussian) distribution. The Spearman correlation coefficient (*r*_*s*_) was calculated to quantify the relationship between treatment history indicators and indicators of loiasis endemicity. Contingency tables, with chi-square tests, and the chi-square test for trends, were used to assess the association between the frequency of individuals in the different ivermectin treatment groups and age/sex.

The mixed effects logistic regression model was used to explore whether ivermectin intake was a significant factor in *L*. *loa* mf prevalence and the risk of harbouring high/very high mf intensities. The CDTI projects were considered random effects while fixed effects were the covariates: age (children or adults, categorical), sex (males or females, categorical), ivermectin treatment (never, 1–5 times or > 5 times, categorical). The outcome variables were either the proportion of individuals with zero mf or the proportion of individuals with mf below 8000mf/ml or the proportion of individuals with mf load between 8001-30000mf/ml or proportion of individuals with >30,000 mf/ml. Reference categories for our fixed effects were: adults, females and individuals who had never taken ivermectin respectively. The Odd Ratios (ORs) indicated the magnitude and direction of the ivermectin treatment, age or sex effect for the various ivermectin treatment history/age/sex groups. If ivermectin intake, age and sex demonstrated a protective effect against harbouring mf, or high or very high mf loads or no mf, the ORs was less than 1 and statistically significant. All the statistical tests were performed at a 5% significance level.

## Results

### Study population characteristics

**Fig 2:**
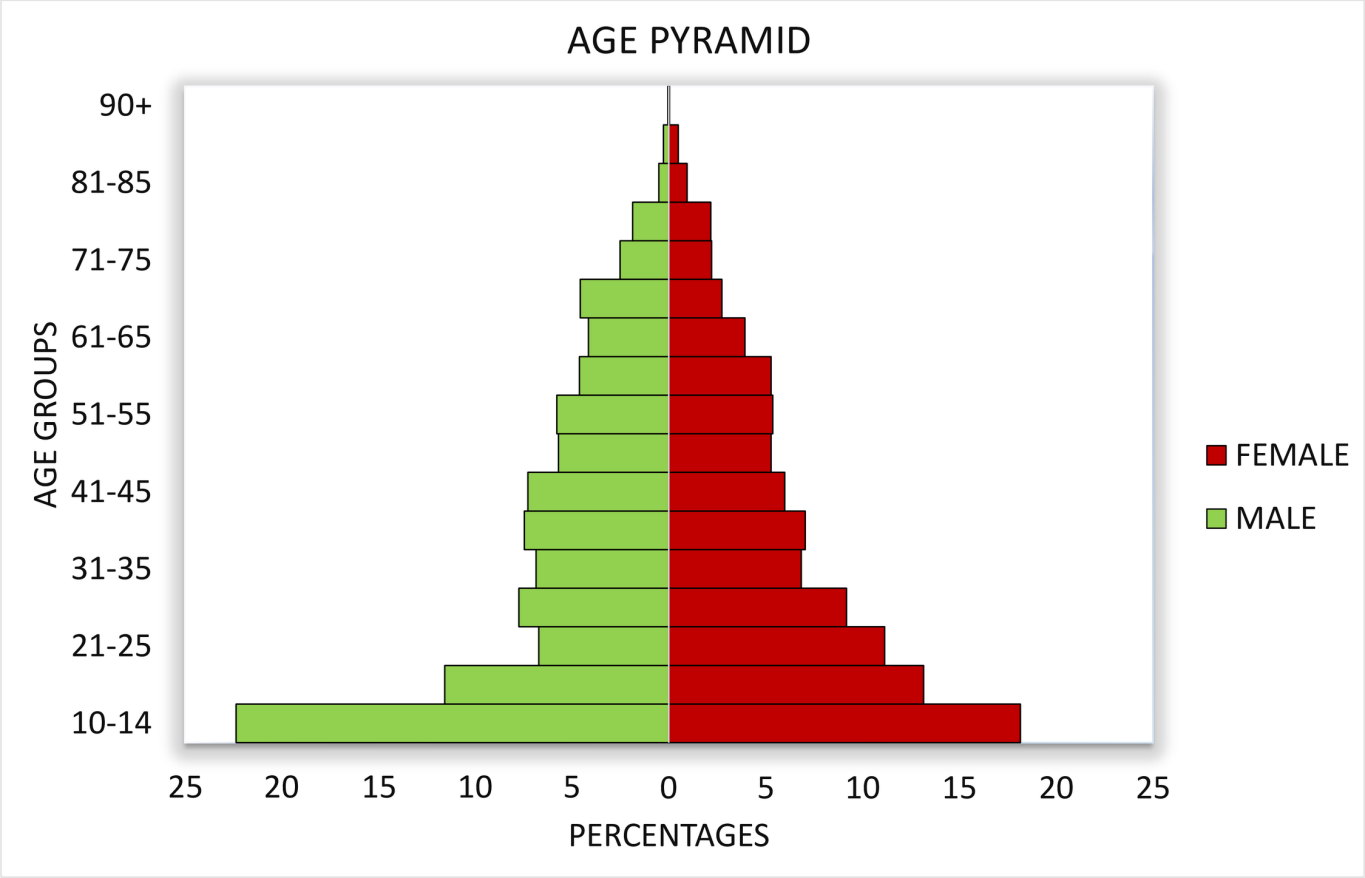
Pyramid of ages of males and females of the study population.

A total of 3,684 individuals (54% female) were recruited from 36 communities of the 3 CDTI project sites, while 900 (48% female) came from the 9 non-CDTI communities (Table 1). Age varied from 10–95 years (Fig 2).

**Table 1:** Number of communities surveyed and number of individuals examined in the various study sites.

PROJECT SITE		Number of Communities		Gender	
	Age Group		Number Examined	Male	Female
**EAST**	Children	16	293	153	140
	Adults		1130	527	603
	Subtotal		1423	680	743
**NORTHWEST**	Children	10	240	108	132
	Adults		1089	419	670
	Subtotal		1329	527	802
**SOUTHWEST**	Children	10	199	114	85
	Adults		733	356	377
	Subtotal		932	470	462
**TOTAL CDTI**		**36**	**3684**	**1677**	**2007**
**EAST Non-CDTI**	Children	9	190	104	86
	Adults		710	363	347
	Subtotal		900	467	433
**Grand Total**		**45**	**4584**	**2144**	**2440**

### Prevalence and intensity of *L*. *loa* mf before and after several rounds of CDTI

#### East project site

**Fig 3:**
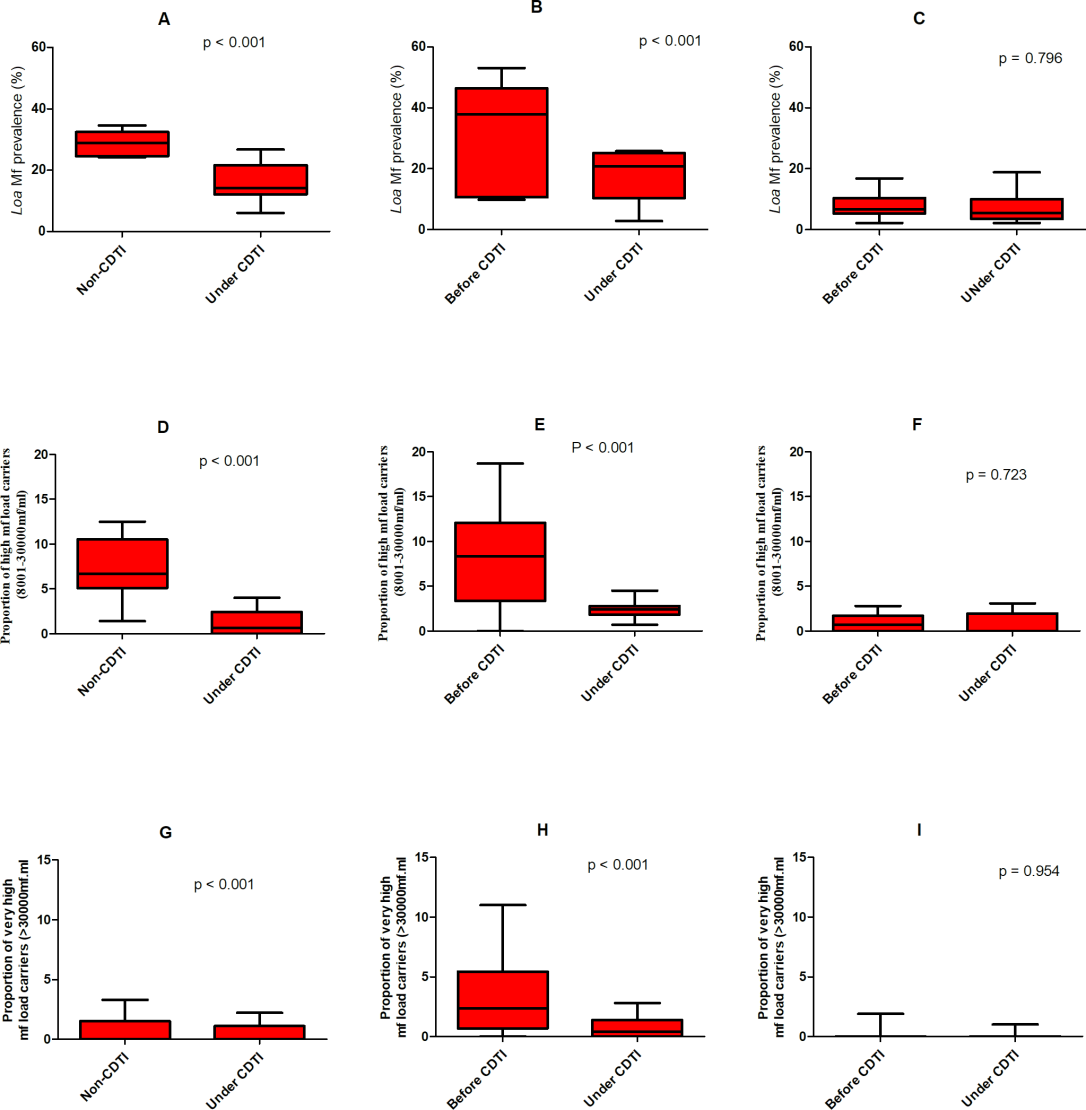
**A-C: Box and whiskers plots of *L*. *loa* microfilarial prevalence in the adult population of the East, Northwest and Southwest 2 CDTI project sites.** For the East project, baseline data were not available, but data from a geographically close non-CDT district are shown A) East sites. Left plot presents the results for the non-CDTI communities (n = 710), with median mf prevalence of 31.30% (minimum = 24%; maximum = 35%, 95% CI = 29.05–29.55); the plot on the right corresponds to 10 years of CDTI (n = 1130), with median prevalence = 15.2% (min = 3%; max = 27%, 95% CI = 16.00–16.52). B) In the Northwest site (n = 1028, 9 years of CDTI), the median prevalence on the left (baseline plot) is 34.6% (min = 10%; max = 53%, 95% CI = 29.5–31.5%) at baseline; the plot on the right (n = 1089, 9 years of CDTI) depicts a median prevalence of 19.3% (min = 2.8%; max = 35%, 95% CI = 17.8–18.8%). C) In the Southwest 2 project, the median prevalence of the left (pre-ivermectin, n = 1458) plot is 7% (min = 2.2%; max = 16.8%, 95% CI = 7.9–8.3%). Despite 14 years of CDTI (n = 733), the median mf prevalence is 5.4% on the right plot (min = 2.2%; max = 18.8%, 95% CI = 7–7.7%). The p-values shown correspond to the appropriate tests to compare 2-sample proportions applied to the overall mf prevalence (see main text), **D-E: Box and whiskers plots of the prevalence of individuals with high *L*. *loa* microfilarial loads (8,001–30,000mf/ml) in the adult population of the East, Northwest and Southwest 2 CDTI project sites.** For the East project, baseline data were not available, but data from a geographically close non-CDT district are shown albeit not formally compared. G) East sites. Left plot presents the results for the non-CDTI communities, with median high load mf prevalence of 6.7% (minimum = 1.4%; maximum = 12.5%, 95% CI = 4.5–9.8%); the plot on the right corresponds to 10 years of CDTI, with median prevalence = 0.7% (min = 0%; max = 4%, 95% CI = 0.5–2.1%). H) In the Northwest site (9 years of CDTI), the median prevalence on the left (baseline plot) is 8.4% (min = 0%; max = 18.7%%, 95% CI = 4.2–12.2%) at baseline; the plot on the right (9 years of CDTI) depicts a median prevalence of 2.4% (min = 0.7%; max = 4.5%, 95% CI = 1.6–3.1%). I) In the Southwest 2 project, the median prevalence of the left (pre-ivermectin) plot is 0.7% (min = 0%; max = 2.8%, 95% CI = 0.4–1.4%). Despite 14 years of CDTI, the median mf prevalence is <0.00001% on the right plot (min = 0%; max = 3.1%, 95% CI = -0.2–1.8%). The p-values shown correspond to the appropriate tests to compare 2-sample proportions applied to the overall mf prevalence (see main text) and **G-I Box and whiskers plots of the prevalence of individuals with very high *L*. *loa* microfilarial loads (>30,000mf/ml) in the adult population of the East, Northwest and Southwest 2 CDTI project sites.** For the East project, baseline data were not available, but data from a geographically close non-CDTI district are shown albeit not formally compared. D) East sites. Left plot presents the results for the non-CDTI communities, with median very low load mf prevalence of <0.00001% (minimum = 0%; maximum = 3.3%, 95% CI = -0.1–1.7%); the plot on the right corresponds to 10 years of CDTI, with median prevalence = <0.00001% (min = 0%; max = 2.2%, 95% CI = 0.1–0.9%). E) In the Northwest site (9 years of CDTI), the median prevalence on the left (baseline plot) is 2.4% (min = 0%; max = 11%, 95% CI = 0.8–6.1%) at baseline; the plot on the right (9 years of CDTI) depicts a median prevalence of 0.4 (min = 0%; max = 2.8%, 95% CI = 0.01–1.6%). F) In the Southwest project, only the whiskers are plotted (median and interquartile values were equal to zero), with min = 0%; max = 1.9% for the baseline data, and with min = 0% and max = 1% after 14 years of CDTI. The p-values shown correspond to the appropriate tests to compare 2-sample proportions applied to the overall mf prevalence (see main text).

In the East region, there were no baseline infection data for the 16 CDTI villages included in the study. Although a formal comparison cannot be made, data from the 9 non-CDTI communities are informative and only data from adults were used for this analysis. All surveyed communities in the East non-CDTI district were highly endemic for *L*. *loa*, with an overall mf prevalence of 29.3% (n = 710; 95% CI = 26.0–32.8%) in the adult population (ranging from 24.2% to 34.6% among the communities). These prevalence values contrast with those observed in the CDTI sites after 10 years of treatment (overall = 16.0%; n = 1,130; 95% CI = 13.9–18.3%), which ranged from 3.3% to 26.6% (Fig 3A). The arithmetic mean infection intensity in the non-CDTI district (1,727 mf/ml) was 2.6 times as high as that recorded in the CDTI sites (670 mf/ml, U = 10.0, p = 0.0002).

#### Northwest project site

The 10 communities of this project site had baseline parasitological data on the adult population, so these pre-control data were compared to the recently generated ones after 9 rounds of CDTI. The results indicate that after 9 years of ivermectin treatment, the epidemiological situation in most communities had fundamentally changed. The overall mf prevalence post-CDTI of 17.9% (n = 1,089; 95% CI = 15.7–20.3%), ranging from 2.8% to 34.9% among the sampled villages, was significantly lower than the pre-control prevalence of 30.5% (n = 1,028; 95% CI = 27.7–33.5%), ranging from 9.9% to 53.1%) as depicted in Fig 3B (z = 6.8008, p<0.0001). The trend in mf intensity was similar to that of mf prevalence, with the pre-control arithmetic mean of 2,993.5 mf/ml being significantly higher than the post-control mean of 1,118.9 mf/ml (U = 24.0, p = 0.0090).

#### Southwest 2 project site

In the South West region, where baseline data were also available for adults, the *L*. *loa* endemicity of most communities did not drastically shift between the pre-control and the evaluation periods after 14 years of CDTI. The 10 communities visited during the evaluation phase remained moderately endemic. The overall mf prevalence in the evaluation phase of 7.8% (n = 733; 95% CI = 5.9–10.0%; range = 2.2% to 18.8%) was not significantly different from that at pre-control of 8.1% (n = 1,458; 95% CI = 6.8–9.6%; range = 2.2% to 16.8%, p = 0.796, X^2^ = 0.067), as shown in Fig 3C. However, the pre-control mf intensity of 343.8 mf/ml was statistically different from the post-control intensity of 213.2 mf/ml (U = 51.0, p = 0.024).

### Prevalence of individuals with high *L*. *loa* mf load (8,001–30,000 mf/ml)

In the East study sites, the prevalence of individuals with high microfilaraemia (among adults), after 10 years of CDTI, ranged from 0 to 4%, with a general prevalence of 1.2% (n = 1130; 95% CI = 0.7–2.1%). This was markedly lower than the prevalence obtained from non-CDTI communities (6.2%; n = 710; 95% CI = 4.5–8.2%), where it ranged from 1.4 to 12.5% (Fig 3D). In the Northwest site, and after 9 years of CDTI, the overall prevalence of individuals with high mf loads was 2.3% (n = 1,089; 95% CI = 1.6–3.4%) and ranged from 0.7% to 11.6% between the villages of this project. This was statistically significantly lower than the overall pre-control value of 7.7% (n = 1,028; 95% CI = 6.1–9.5%), ranging from 0% to 18.7% among the communities (X^2^ = 31.115, p <0.0001, (Fig 3E). In the Southwest 2 project site, the overall prevalence of high mf load carriers post 14 years of CDTI was 0.7% (n = 733; 95% CI = 0.2–1.6%), with villages ranging from 0% to 3.1%, not experiencing significant change from the pre-control value of 0.8% (n = 1,458; 95% CI = 0.4–1.4%), with range from 0% to 2.8% (X^2^ = 0.126, P = 0.723, (Fig 3F).

### Prevalence of individuals with very high *L*. *loa* mf load (>30,000 mf/ml)

In the East study sites, the prevalence of individuals with very high microfilaraemia (among adults), after 10 years of CDTI, ranged from 0 to 2.2%, with a general value of 0.62% (n = 1,130; 95% CI = 0.25–1.3%). This was not different from the prevalence obtained from non-CDTI communities (1%; n = 710; 95% CI = 0.4–2%), where it ranged from 0 to 3.3%, z-value = 0.9, p-value (two-tailed test) = 0.375 (Fig 3G). In the Northwest site, and after 9 years of CDTI, the overall prevalence of individuals with very high mf loads was 0.8% (n = 1,089; 95% CI = 0.4–1.6%) and ranged from 0% to 3.5% between the villages of this project. This was statistically significantly lower than the overall pre-control value of 3.3% (n = 1,028; 95% CI = 2.3–4.6%), ranging from 0% to 11% among the communities; z-value = 4, p-value (one-tailed test) < 0.0001 (Fig 3H). In the Southwest 2 project site, the overall prevalence of very high mf load carriers post 14 years of CDTI was 0.11% (n = 932; 95% CI = 0–0.6%) in the general population, with villages ranging from 0% to 1%, not changing significantly from the pre-control value of 0.2% (n = 1,458; 95% CI = 0.04–0.6%), with range from 0% to 1.9%; chi-squared = 0.003 (1 DF), p-value = 0.954 (Fig 3I).

### Oral declaration of ivermectin intake

Treatment coverage was ascertained in all 36 surveyed CDTI communities based on questionnaires. Treated individuals were able to recall the number of times they had taken ivermectin, which ranged from 1 to > 10 times. The study results indicated that over 80% of adult respondents interviewed had participated in the ivermectin MDA at least once, although >50% of these individuals were low compliers (had taken ivermectin < 5 times). In the East, Northwest and Southwest project sites, 21%, 29% and 17% of the adult population, respectively, declared to have never taken ivermectin treatment. In the East study sites, there was a significant difference across the different treatment groups in adults and children and between adult males and females. The same trend was observed in the Northwest and Southwest project sites except for the fact that there was no significant difference in treatment intake between adult males and females (Table 2). Taking the three CDTI projects as a whole, most treated adult individuals declared to have taken ivermectin 1–2 times (27.3%), while relatively few (6.3%) were treated more than 10 times. (The duration of the CDTI project since inception till the time of the study ranged from 9 to 14 years; see above.)

**Table 2:** Oral declaration of ivermectin intake in the CDTI project sites with respect to sex and age group.

	Age Group	Gender	Ivermectin Treatment Classes							Total	P-value
			0	1–2	3–4	5–6	7–8	9–10	11+		
EAST	Children	Male	23 (17.6)	64 (48.9)	29 (22.1)	9 (6.9)	6 (4.6)			131	< 0.0001*
											0.9084**
		Female	22 (18.2)	55 (45.5)	32 (26.4)	9 (7.4)	3 (2.5)			121	
		Subtotal	45 (17.9)	119 (47.2)	61 (24.2)	18 (7.1)	9 (3.6)			252	
	Adults	Male	82 (19.4)	100 (23.7)	102 (24.2)	48 (11.4)	90 (21.3)			422	0.0126*
											<0.0001**
		Female	107 (23.2)	116 (25.2)	108 (23.4)	43 (9.3)	87 (18.9)			461	
		Subtotal	189 (21.4)	216 (24.5)	210 (23.8)	91 (10.3)	177 (20.0)			883	0.0030***
NORTHWEST	Children	Male	53 (49.5)	37 (34.6)	16 (15.0)	1 (0.9)				107	< 0.0001*
											0.0412**
		Female	53 (40.2)	43 (32.6)	30 (22.7)	6 (4.5)				132	
		Subtotal	106 (44.4)	80 (33.5)	46 (19.2)	7 (2.9)				239	
	Adults	Male	99 (23.7)	148 (35.4)	111 (26.6)	44 (10.5)	16 (38)			418	< 0.0001*
											0.1416**
		Female	220 (33.0)	228 (34.2)	139 (20.8)	70 (10.5)	10 (1.5)			667	
		Subtotal	319 (29.4)	376 (34.7)	250 (23.0)	114 (10.5)	26 (2.4)			1085	0.0029***
SOUTHWEST	Children	Male	38 (33.3)	37 (32.5)	28 (24.6)	9 (7.9)	2 (1.8)			114	< 0.0001*
											0.0237**
		Female	36 (42.4)	31 (36.5)	15 (17.6)	3 (3.5)	0 (0)			85	
		Subtotal	74 (37.2)	68 (34.2)	43 (21.6)	12 (6.0)	2 (1.0)			199	
	Adults	Male	53 (15.0)	70 (19.8)	85 (24.1)	79 (22.4)	13 (3.7)	17 (4.8)	36 (10.2)	353	< 0.0001*
											<0.1096**
		Female	78 (20.7)	96 (25.5)	80 (21.2)	65 (17.2)	19 (5.0)	21 (5.6)	18 (4.8)	377	
		Subtotal	131 (17.9)	166 (22.7)	165 (22.6)	144 (19.7)	32 (4.4)	38 (5.2)	54 (7.4)	730	<0.0001***

*Chi square test for trend of differences in percentages between IVM treatment classes

** Chi square test for trend of differences in percentages of compliance between male and female per study site

***Chi square test for trend of differences in percentage of compliance between adults and children.

Values in brackets are percentages

### Relationship between adherence to ivermectin treatment and microfilarial prevalence of *L*. *loa* infection

#### Treatment history indicators

**Fig 4:**
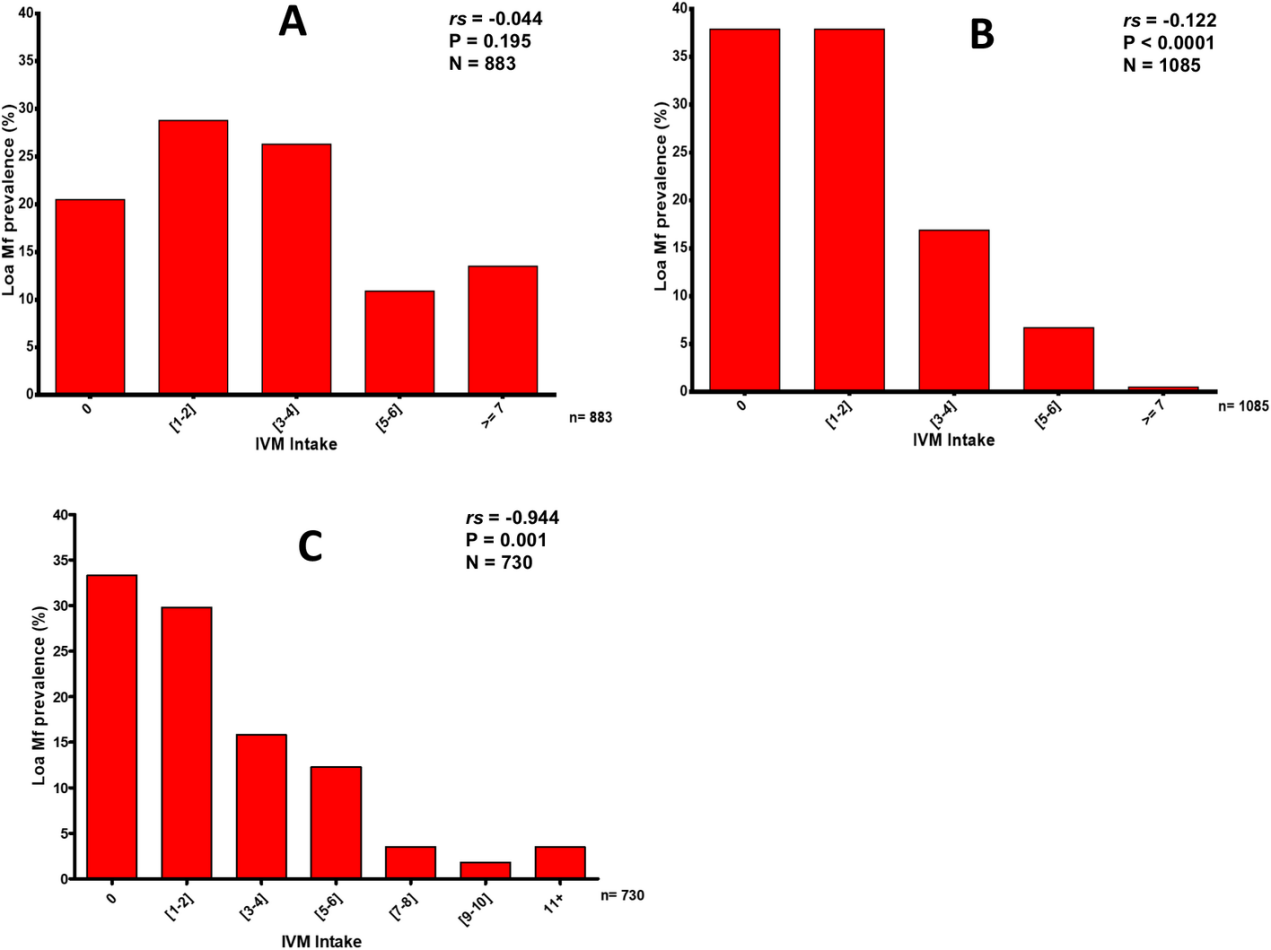
Relationship between *Loa loa* microfilarial prevalence and ivermectin intake in the East, Northwest and Southwest CDTI project sites. A) In the East, an increase in the number of treatment rounds led to a decrease in *L*. *loa* mf prevalence although with a non significant difference. B) In the Northwest, *L*. *loa* mf prevalence inversely correlated with ivermectin treatment rounds with a very significant difference. C) The same relationship was depicted in the Southwest where a decrease in *L*. *loa* mf prevalence was related to an increase in number of ivermectin treatment rounds with a significant difference. The *r*_*s*_, p values and sample sizes are presented on the figures.

Study participants treatment histories were correlated with the prevalence of *L*. *loa* infection in the different study sites. Fig 4 depicts the relationship between declared MDA adherence and mf prevalence after 10 mass drug distributions in the East, 9 in the Northwest and 14 in the Southwest 2 CDTI projects. People who reported that they had never taken ivermectin had a higher mf prevalence than those who reported that they had taken more than 4 ivermectin doses. Generally, an increase in number of treatments led to a decrease in mf prevalence within the study population and the difference was significant in all except the East study sites.

### Relationship between adherence to ivermectin treatment and the prevalence of individuals with different classes of microfilarial intensity of *L*. *loa* infection

#### Treatment history indicators

**Fig 5:**
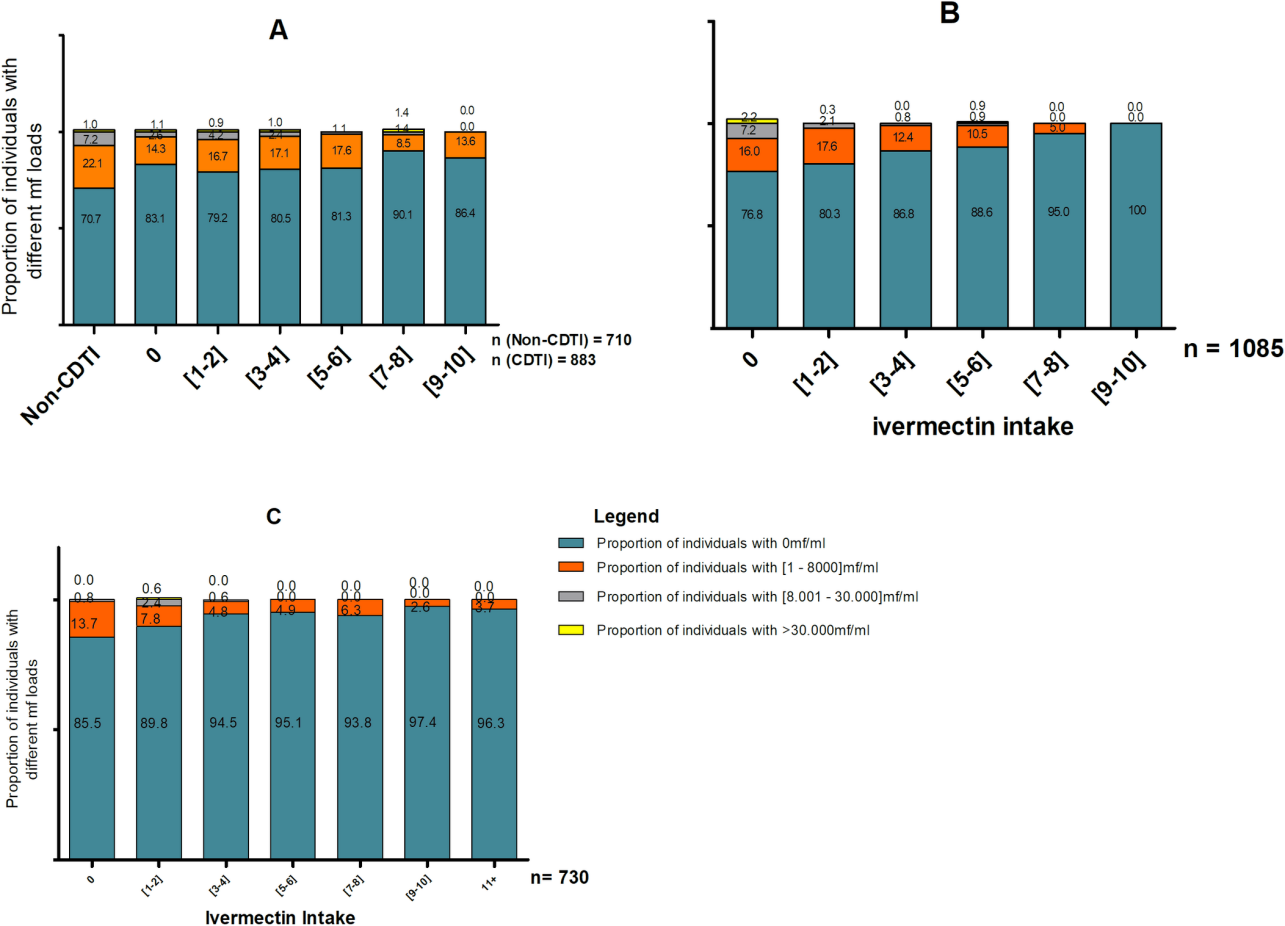
Relationship between the percentage of individuals in different *Loa loa* microfilarial load classes and ivermectin intake in the three CDTI projects. A) In the East, an increase in treatment adherence led to a decrease in the proportion of individuals with high and very high mf loads. B) in the Northwest, the same relationship was depicted and here no individual with >7 treatment rounds was found to have high or very high mf loads. C) in the Southwest, individuals with > 5 treatment rounds were not found to have high or very high mf loads. The sample sizes are indicated on the figures.

As observed with prevalence, there was also a generally positive impact of CDTI on the proportion of individuals with high and very high mf loads (Fig 5). An increase in treatment adherence led to a decrease in the proportion of individuals with high and very high mf loads. Within low (treatment taken 1–4 times) and non-compliers (never taken treatment), there existed individuals with high and very high mf loads, while within high compliers (those who had taken ivermectin >5 times), very few or no individuals were found to have high or very high mf loads This clearly demonstrates that the more an individual adheres to ivermectin treatment, the less likely he/she is to harbour high or very high mf loads.

### Percentage reduction of parasitological indicators of loiasis before and under CDTI in the study sites

The proportions of high mf load carriers were significantly reduced in the Northwest and Southwest sites and different in the East while those of the very high mf load carriers were significantly reduced in the Northwest and different in the East. In the Southwest site, no difference was observed between the two study periods as observed in Table 3. The proportion of high mf load carriers was reduced by 73.6%, 59.7% and 20% in the East, Northwest and Southwest sites respectively while the proportion of very high mf load carriers was reduced by 40%, 75.8% and 50% in the respective sites. Amongst children of the East region, there were none with high or very high mf load following 10 years of ivermectin treatment (nonCDTI n = 190, CDTI n = 293, Table 4).

**Table 3:** Percentage reduction/difference* of the prevalence of individuals with *Loa loa* microfilariae (overall mf prevalence) and of individuals within categories of mf intensity before and under CDTI in the study sites.

Project	Indicators	Before CDTI/No CDTI	Under CDTI	% reduction	p-value
East*	Mf prevalence	29.3	16	45.4	< 0.0001
	[1–8,000]mf/ml	22.1	14.2	35.7	< 0.0001
	8,001–30,000mf/ml**	7.2	1.9	73.6	< 0.0001
	> 30,000mf/ml***	1.0	0.6	40.0	< 0.0001
Northwest	Mf prevalence	30.5	17.9	41.3	< 0.0001
	[1–8.000]mf/ml	22.9	14.8	35.4	< 0.0001
	8,001–30,000mf/ml*	7.7	3.1	59.7	< 0.0001
	> 30,000mf/ml**	3.3	0.8	75.8	< 0.0001
Southwest 2	Mf prevalence	8.1	7.8	3.7	0.796
	[1–8,000]mf/ml	7.1	7.0	1.4	0.927
	8001–30.000mf/ml *	1.0	0.8	20.0	0.723
	> 30,000mf/ml**	0.2	0.1	50.0	0.720

*In the East project site, percentage differences were measured and not reductions since we didn’t have baseline data here. “Before CDTI” in this site represents the “non-CDTI” site while “Under CDTI” represents the “CDTI site”.

**Prevalence of individuals at risk of non-neurological complications post ivermectin treatment

***Prevalence of individuals at risk of neurological complications post ivermectin treatment

**Table 4:** Prevalence of children (aged 10–14 years) with Loa loa microfilariae (overall mf prevalence) and of children within categories of mf intensity the East study sites.

Project	Indicators	Non-CDTI district (%)	CDTI district, 10 yr MDA (%)
East	Mf prevalence	5.3 (95% CI = 7.6–9.5%)	3.4 (95% CI = 1.7–6.2%)
	[1–8,000]mf/ml	3.7 (95% CI = 1.5–7.4%)	3.4 (95% CI = 1.7–6.2%)
	8,001–30,000mf/ml *	1.1 (95% CI = 0.1–3.8)	0.0 (95% CI = 0)
	> 30,000mf/ml**	0.5 (95% CI = 0–2.9%)	0.0 (95% CI = 0)

*Prevalence of individuals at risk of non-neurological complications post ivermectin treatment (see comments above)

**Prevalence of individuals at risk of neurological complications post ivermectin treatment

### The Association between adherence to ivermectin, sociodemographic factors and the risk of harbouring Zero, low, high and very high mf loads in the study population

Logistic regression analysis revealed that individuals who had taken ivermectin 5 times or more had an OR of 2.1. This means that they are close to two times more likely to have no mf compared to the zero ivermectin intake group (95% CI [1.5–2.9], p < 0.0011; Table 5). Individuals in the general population who had undergone at least 5 treatment rounds had significantly reduced the risks of getting either low (1-8000mf/ml), high (8001-30000mf/ml) or very high (>30000mf/ml) mf loads (Table 5). Furthermore, age and sex were significant factors which could predict zero or low mf loads as adults and males were those with higher odds of having zero and low mf loads. Surprisingly, age and sex were no longer significant predictors of high and very high mf loads while only ivermectin intake remained the most significant predictor to high and very high mf loads (Table 5).

**Table 5:** The association between adherence to ivermectin, sociodemographic factors and the risk of harbouring zero, low, high and very high mf loads in the study population.

Outcome Variables	Characteristics of the Model	Variables	Examined	Positive^a^	OR	CI 95%		P value
						Lower	Upper	
Odds of having zero mf/ml	Age Group	Children	690	667	Ref			
		Adults	2698	2290	0.172	0.112	0.266	<0.0001
	Gender	Female	1800	1627	Ref			
		Male	864	1330	0.717	0.582	0.883	0.002
	Adherence	0	864	731	Ref			
		1–4	1800	1568	1.293	1.020	1.639	0.034
		≥ 5	724	658	2.088	1.506	2.894	<0.0001
Odds of having individuals with mf between 1-8000mf/ml (Low mf load)	Age Group	Children	690	23	Ref			
		Adults	2698	347	4.672	3.025	7.217	<0.0001
	Gender	Female	1800	183	Ref			
		Male	864	187	1.395	1.118	1.740	0.003
	Adherence	0	864	104	Ref			
		1–4	1800	203	0.891	0.689	1.151	0.377
		≥ 5	724	63	0.614	0.436	0.865	0.005
Odds of having individuals with mf between 8001-30000mf/ml (High mf load)	Age Group	Children	690	0	Ref			
		Adults	2698	44	3.5^10^8^	0.000	-	0.996
	Gender	Female	1800	25	Ref			
		Male	864	19	1.093	0.603	1.981	0.734
	Adherence	0	864	20	Ref			
		1–4	1800	23	0.520	0.286	0.945	0.036
		≥ 5	724	1	0.045	0.006	0.327	0.008
Odds of having individuals with mf > 30000mf/ml (Very high mf load)	Age Group	Children	690	0	Ref			
		Adults	2698	17	1.2^10^8^	0.000	-	0.995
	Gender	Female	1800	8	Ref			
		Male	864	9	1.630	0.842	3.155	0.124
	Adherence	0	864	9	Ref			
		1–4	1800	6	0.293	0.143	0.598	0.005
		≥ 5	724	2	0.194	0.067	0.558	0.008

^a^Number of individuals identified in each mf load group with respect to the variables of interest

### *Loa loa* prevalence in children

A total of 922 children were screened during this study and the results indicated that the Northwest had the highest *L*. *loa* mf prevalence with 5.4% while the East non CDTI, East CDTI and Southwest respectively recorded 5.3%, 3.4% and 1.0%. Important to note is that almost three quarters (71.5%) of the children had either never taken the drug or had taken it less than 3 times during the previous MDAs (Table 2). High and very high mf load carriers were registered only in the non-CDTI site while in the areas under MDA, no child was found to have a high or very high mf load (Table 6).

**Table 6:** Percentage of children with defined mf load of *L*. *loa* before and under CDTI at different project sites.

		[0 mf/ml]		[1–8,000 mf/ml]		[8,001–30,000 mf/ml]		[>30,000 mf/ml]	
Study site	Age group	Before CDTI (%)	After CDTI (%)	Before CDTI (%)	After CDTI (%)	Before CDTI (%)	After CDTI (%)	Before CDTI (%)	After CDTI (%)
East*	Children	180 (96.3)	283 (96.6)	7 (3.7)	10 (3.4)	2 (1.1)	0	1 (0.5)	0
Northwest	Children		227 (94.6)		13 (5.4)		0		0
Southwest	Children		197 (99.0)		2 (1.0)		0		0

The “Before CDTI” in the East represents the non-CDTI region.

Values in brackets are percentage

## Discussion

After 9 to 14 years of CDTI in onchocerciasis-loiasis co-endemic areas in Cameroon, the prevalence and intensity of loiasis have decreased, particularly in those areas that had a higher initial endemicity. One important objective of this study was to assess the impact of CDTI on the parasitological indicators of loiasis. Several studies have demonstrated the excellent efficacy of ivermectin on the microfilaraemia of *L*. *loa* [8, 11, 13, 14, 16, 17]. These previous observations were from clinical or community trials efficacy studies. This is the first time the impact on the parasitological indicators of loiasis of large-scale, repeated annual ivermectin treatment in a programmatic context has been reported. An important feature of the present study is the fact that the repeated annual MDA of ivermectin was initially designed to control onchocerciasis, not loiasis. Because of the co-occurrence of the two filarial species in the study area, we seized this unique opportunity to assess the effect of CDTI on the prevalence and intensity of loiasis. The fact that there exists a group of individuals who systematically refuse to take ivermectin (non-adherents) remains a major stumbling block to the CDTI strategy on both onchocerciasis and loiasis control. Strategies aimed at improving adherence to ivermectin are discussed later.

These cross-sectional surveys have clearly demonstrated a marked reduction and difference in the prevalence of *L*. *loa* in the Northwest and East CDTI projects respectively despite contrasting pre-control endemicity levels of infection of over 30%. The Southwest 2 CDTI project did not experience a significant reduction in *L*. *loa* prevalence. It should be noted that the pre-control prevalence of *L*. *loa* in this project was below 10%. the same trend of reduction in prevalence of infection was also observed in intensity where a significant drop and difference in the microfilaraemia intensity could be observed in the Northwest and East respectively. In the two CDTI projects with higher pre-control endemicity (>20%), of loiasis (East and Northwest), corresponding to more than 5% of the sampled population with 8001-30000mf/ml *L*. *loa* mf/ml, the percentage of these individuals with high intensity of infection reduced to less than 2%. A reduction though not significant in the proportion of individuals with high intensity of *L*. *loa* infection was also observed in the Southwest 2 CDTI project site. Similar changes were observed in the proportions of individuals with very high microfilaria loads of *L*. *loa* (>30000 mf/ml of blood) with significant reduction in the Southwest and Northwest and a difference in the East noted.

One striking and perhaps surprising finding of this study regarding the effect of CDTI on the parasitological indicators of loiasis is the fact that despite the net reduction/difference in the prevalence and intensity of infection in these communities under 9–14 years of CDTI, the proportion of people still harbouring *L*. *loa* infection was quite substantial. Contrary to onchocerciasis, for which mathematical models exist that predict the impact of repeated annual ivermectin treatment on the parasitological and entomological indicators of transmission [33, 34], no such models of prediction exist yet for *L*. *loa* [35]. We do not, therefore, have information on what the expected infection levels should be after a decade or more of ivermectin MDA with given levels of coverage and adherence. But one thing is sure, the infection levels in the residents of these communities are still relatively high. Given the fact that in the clinical and community trials it was demonstrated that a single dose of ivermectin can lead to 80–90% reduction of *L*. *loa* microfilaraemia [15, 16], one could have expected to have a lower fraction of individuals still harbouring *L*. *loa* mf. This disappointing observation is well illustrated with the findings from the Southwest 2 CDTI project. Here, the pre-control prevalence was relatively low (less than 10% mf prevalence, with less than 5% of individuals with high *L*. *loa* mf load, and less than 1% of individuals with very high *L*. *loa* mf loads). We could have expected that with more than a decade of repeated annual ivermectin MDA, the parasitological indicators of infection would be very low, offering the possibility of envisaging elimination of *L*. *loa*. However, *L*. *loa* infection was still observed in these communities. Important to note is the fact that during the evaluation phase in the Southwest, the same communities could not be revisited due to inaccessibility of most of the communities. We considered visiting nearby communities of the same bio ecological zone since *L*. *loa* transmission dynamics was the same in this rainforest region [21, 36]. The reductions in the Southwest 2 project site though not significant were most probably CDTI related and not community/geographical setting related.

In an attempt to understand the limited impact of CDTI on *L*. *loa*, we conducted a rapid assessment of adherence to ivermectin treatment using a method previously described in [37]. It was observed that close to 20% of the study population in the East CDTI project declared to have never taken ivermectin treatment despite 10 years of annual distribution. The situation was even more worrisome in the Northwest CDTI project, with close to 35% of individuals declaring never taking the treatment. The Southwest 2 CDTI project also recorded close to 25% of individuals declaring being systematically non-adherent to the annual treatments. If one adds to these proportions of systematic non-adherence to ivermectin treatment the proportion of those who took the drug less than 5 times, one gets more than 50% of the population who are in the low compliance or systematic non-compliance categories. Similar observations were made recently for onchocerciasis in Southwest Cameroon [38]. The fundamental question is to try to understand why the population of these areas of co-endemicity between onchocerciasis and loiasis are so reticent to taking ivermectin. Several studies have documented the side effects associated to ivermectin treatment in *L*. *loa*-infected patients, including SAEs [19, 39–41]. By 2015, more than 400 SAEs had been reported during control of onchocerciasis with ivermectin MDA in *L*. *loa* co-endemic areas. Despite the fact that the joint Expert Committee of APOC and the Mectizan Donation Programme (MDP) developed a guide for implementation of ivermectin treatment of onchocerciasis in areas of co-endemicity with loiasis [20] (a treatment guide that emphasizes the prompt detection and management of the adverse events to prevent their severity and eventual fatal outcome), the psycho-social impact of the SAEs has led to a perceived fear of severe reactions; and this could have been a major obstacle to ivermectin adherence [38, 42].

Findings from the present study clearly indicate a negative association between ivermectin intake and *L*. *loa* mf prevalence. This association was statistically significant for the CDTI projects of the Northwest and Southwest. A *L*. *loa* mf prevalence above 30% could still be observed in people who declared to have never taken ivermectin, or who took it less than 3 times, with the mf prevalence dropping drastically in those who complied 5 times and more. These results are in agreement with observations made recently on the relationship between adherence to ivermectin and parasitological indicators of onchocerciasis [37]. There was also a negative association between the number of times individuals took ivermectin and their intensity of *L*. *loa* microfilaraemia. Essentially, individuals harbouring very high *L*. *loa* mf loads (>30,000 mf/ml) were seen only in the group of individuals who either systematically refused to take the treatment, or took it less than 5 times. Again, similar observations were made between adherence to ivermectin treatment and onchocerciasis infection intensity [37]. The fact that a substantial proportion of inhabitants in these forest areas co-endemic for loiasis and onchocerciasis does not adhere well to ivermectin MDA, and this proportion of individuals are likely to harbour both *O*. *volvulus* and *L*. *loa* with presumably high infection intensities poses a real problem for the perspectives of eliminating onchocerciasis in these areas of co-endemicity.

The relationship between ivermectin and *L*. *loa* presents an intriguing vicious cycle that was illustrated recently with the development of an animal model of *L*. *loa* encephalopathy [15, 43]. Ivermectin is highly efficacious in clearing the *L*. *loa* mf from the peripheral blood system; this clearance is associated with the rapid killing of *L*. *loa* mf in the body tissues. The obstruction of blood capillaries by these dead worms in the organs leads to an ischemic reaction, increased pressure within capillaries, rupture of the affected vessel(s) and haemorrhagic suffusion with consequences that can result in neurological complications, coma and death. The consequences of these two “mutually neutralizing forces” (efficacy of ivermectin against *L*. *loa* mf and associated pathology) are that in *L*. *loa*-endemic areas, the population does not fully benefit from ivermectin as compared to *L*. *loa* non-endemic areas. The major implications of this situation is that onchocerciasis may not be defeated with the current CDTI strategy in areas of *L*. *loa* co-endemicity. Alternative strategies for the treatment of onchocerciasis should be envisaged in these areas if the elimination of onchocerciasis is to be achieved by 2025 as being projected [44]. Among these strategies is the use of therapies that kill *O*. *volvulus* without or with minimal effect on *L*. *loa*, such as anti-Wolbachia antibiotics [45–47] with a test and treat strategy. This could be coupled to a ground larviciding in some areas that are suitable. Another strategy known as the “Test and not Treat” consisting of identifying individuals with relatively high *L*. *loa* mf loads using the LoaScope [48]. The LoaScope is a smartphone-based video-microscope which allows the quantitation of *Loa* microfilaraemia within 2 min after a finger prick. This strategy however, will be more suitable in onchocerciasis hypoendemic areas with *L*. *loa* hyperendemicity [49]. Our data have also demonstrated that individuals with <5 ivermectin treatments (low compliers) are still at risk of developing complications post treatment given that they were still found to harbour high and very high mf loads. Improvements in the programmatic factors like those under the control of the health system and partners will better strategize disease control and subsequent elimination [50].

Avoiding tackling *L*. *loa* by changing the therapy for onchocerciasis may not be a long-term solution for the control and elimination of filariases in *L*. *loa* endemic areas. It was observed recently that *L*. *loa* constitutes a major impediment for the mapping of LF. The Immuno-chromatographic Card Test (ICT) that has been used extensively for the mapping and post-treatment monitoring of LF cross-reacts with *L*. *loa* and, therefore, the initial map of LF in *L*. *loa* endemic areas would need to be revised and updated [51–54]. With these double penalties *L*. *loa* is inflicting to the control of onchocerciasis and LF in the forest areas of the central African region, there is a pressing need of facing the control of this parasite [35]. One long-lasting solution to *L*. *loa* could come from R&D programmes that focus on drugs that can safely be used to treat *L*. *loa* patients and control the infection. Such drug development programme could benefit from the experimental models of *L*. *loa* recently developed (maintenance of different stages in vitro, patent infection in the baboon and inbred mice). Indeed, it was demonstrated recently that Rag C mice could harbour *L*. *loa* patent infections [55]. This finding would be likely to accelerate the development of drugs and diagnostic tools for loiasis. Another strategy to reduce the negative impact of *L*. *loa* for the control of onchocerciasis and LF would be the targeting of its *Chrysops spp*. vectors. These tabanid species present some key vector transmission characteristics that may be targeted for vector control with potential for integrated vector management [56].

The reduction of the potential risk of developing non-neurological and neurological complications in the populations of the CDTI projects seems to be one of the major benefits of ivermectin MDA on *L*. *loa* in areas of co-endemicity with onchocerciasis. Current observations have indicated that the risk of developing marked or serious reactions is significantly higher when *L*. *Loa* mf loads exceed 8,000mf/ml (heavy microfilarial load associated to non- neurological complications); the severity of these reactions worsen in patients with > 30,000 mf/ml, (very high microfilarial loads associated to neurological complications [39, 40]. One striking observation in this study is the net reduction of the proportion of individuals with high and very high mf load (8,001–30,000 and > 30,000 mf/ml of blood). This reduction was highly correlated with the number of times the individuals had taken ivermectin annually. The proportion of individuals with high mf load dropped from 2.6–7.2% in ivermectin-naïve populations (before inception of CDTI) to 0% in those individuals who adhered to ivermectin treatment at least 5 times. A net protective effect of ivermectin MDA on the potential risk of developing non-neurological complications following treatment was observed. The other net benefit was the impact of ivermectin MDA to reduce the potential risk associated with the development of neurological complications due to very high *L*. *loa* mf loads, In the present study, it was observed that the proportion of individuals with very high mf load initially at between 1.1–2.2% could drop to 0–0.6% under regular CDTI, mainly in the group of individuals who adhered to ivermectin treatment more than 5 times. These changes in the proportions of individuals with high and very high *L*. *loa* mf loads can be seen as the potential for averting the risk of developing non-neurological and neurological complications both among CDTI adherents and the general population.

Another striking observation made with this study is the potential protective effect of ivermectin MDA in preventing children from developing high and very high *L*. *loa* mf loads. In the non-CDTI district, contiguous to the one of the East CDTI project sites, 1.1% of children had high mf loads; In the East CDT project no children were recorded as having high or very high mf load (after 10 years of ivermectin MDA). Similar observations were made in the Northwest and Southwest CDTI projects, where no child was found with high or very high mf load (Table 6). These observations could be interpreted as ivermectin MDA reducing the potential risk of children living in CDTI interventions zones for developing non-neurological or neurological complications following ivermectin treatment.

### Conclusion

This study has demonstrated that repeated annual treatment with ivermectin for the control of onchocerciasis using Community directed delivery approach has achieved a significant impact on *L*. *loa* parasitological indicators. This impact was highly felt on the reduction in the proportion of adult individuals with high and very high microfilarial load and with increase in the proportion of the individuals in the study population with no circulating *L*. *loa* microfilariae in their blood. Potentially, the reduction in *L*. *loa* microfilarial intensity could contribute to the prevention of potential risk of individuals who better adhere to ivermectin treatment to develop severe adverse events (SAEs) during subsequent ivermectin mass drug administration campaigns. Children born within CDTi projects are also protected from the potential risk of developing SAEs. The study has revealed that the adherence to the Ivermectin repeated annual treatment is a key determinant for its full impact on *L*. *loa* parasitological indicators. The non-adherence to ivermectin treatment has been demonstrated to be associated to perceived fear of severe adverse events (SAEs). This constitutes an inbuilt design that may prevent ivermectin to ever have full impact on *L*. *loa* transmission in endemic areas. In the areas of co-endemicity *L*. *loa* with *O*. *volvulus* or *Wuchereria bancrofti*, ivermectin would likely also have limited impact on both the parasitological and entomological indicators of those filariae. These observations reinforce the urgent need of putting in place alternative strategies to accelerate the elimination of onchocerciasis and LF in areas co-endemic with *L*. *loa*. This also calls for a research agenda to develop safe drugs to combat *L*. *loa*.

## Supporting information

S1 Fig Flow diagram of the study design. (PDF)

S1 Table Prevalence of defined microfilaria loads before CDTI inception in adults of the different study communities of the 3 regions. (PDF)

S2 Table Prevalence of defined microfilaria loads post CDTI in adults of the different study communities of the 3 CDTI projects. (PDF)

S3 Table Percentage potential risk of non neurological* consequences prevented among CDTI adherents and in the general population of the three CDTI projects. (PDF)

S4 Table Percentage potential risk of neurological* consequences prevented among CDTI adherents and in the population of the three CDTI projects. (PDF)
