# Supplementary material for: Impact of repeated annual community directed treatment with ivermectin on loiasis parasitological indicators in Cameroon: Implications for onchocerciasis and lymphatic filariasis elimination in areas co-endemic with Loa loa in Africa
Source: PLoS Negl Trop Dis. 2018 Sep 18;12(9):e0006750. doi: 10.1371/journal.pntd.0006750 (PMC6161907; doi:10.1371/journal.pntd.0006750)
Supplement: S1 Fig — (PDF) [file pntd.0006750.s001.pdf]

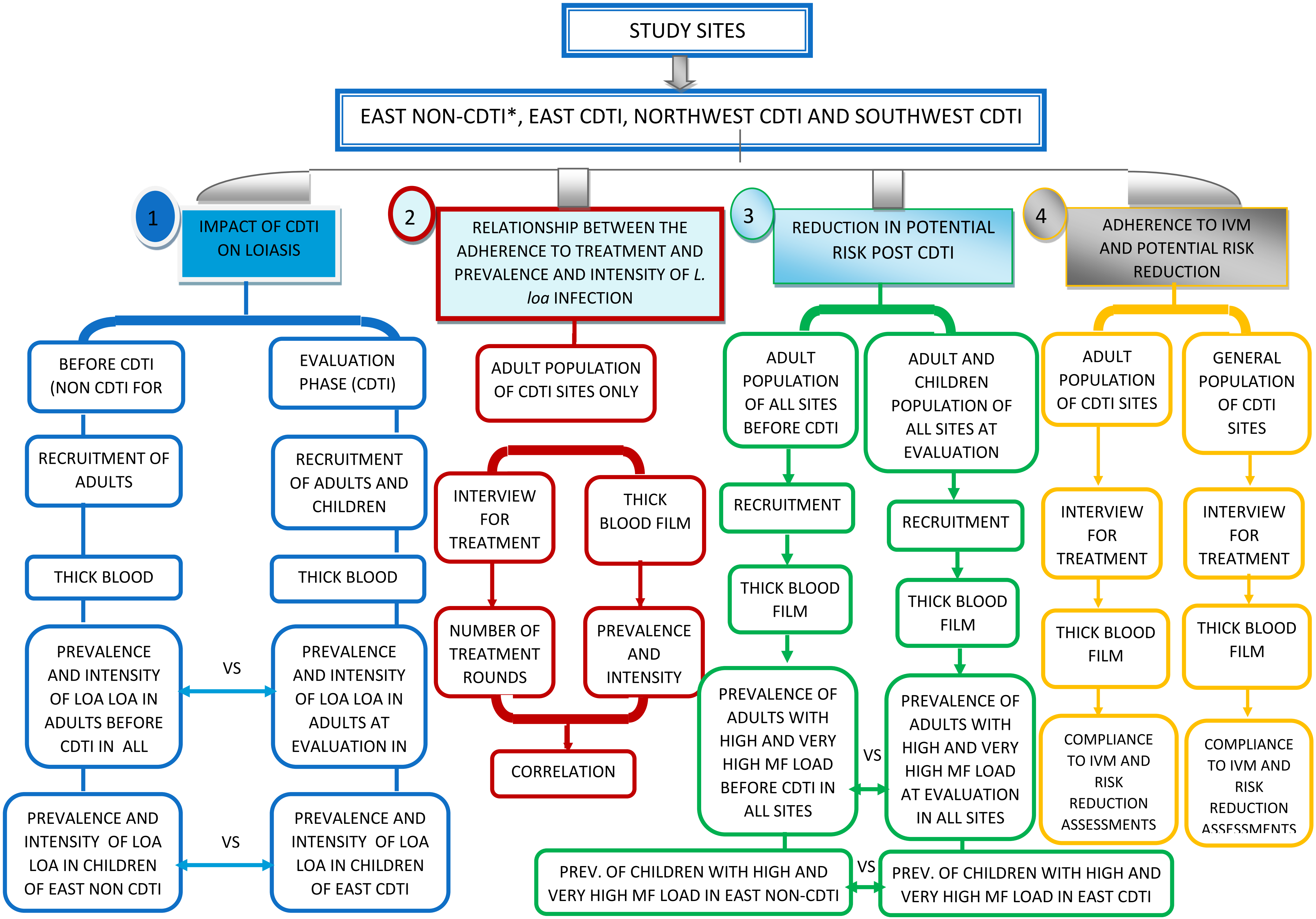
STUDY SITES
EAST NON-CDTI*, EAST CDTI, NORTHWEST CDTI AND SOUTHWEST CDTI
1
IMPACT OF CDTI ON LOIASIS
2
RELATIONSHIP BETWEEN THE ADHERENCE TO TREATMENT AND PREVALENCE AND INTENSITY OF *L. loa* INFECTION
3
REDUCTION IN POTENTIAL RISK POST CDTI
4
ADHERENCE TO IVM AND POTENTIAL RISK REDUCTION
BEFORE CDTI (NON CDTI FOR
EVALUATION PHASE (CDTI)
RECRUITMENT OF ADULTS
RECRUITMENT OF ADULTS AND CHILDREN
THICK BLOOD
THICK BLOOD
PREVALENCE AND INTENSITY OF LOA LOA IN ADULTS BEFORE CDTI IN ALL
VS
PREVALENCE AND INTENSITY OF LOA LOA IN ADULTS AT EVALUATION IN
PREVALENCE AND INTENSITY OF LOA LOA IN CHILDREN OF EAST NON CDTI
VS
PREVALENCE AND INTENSITY OF LOA LOA IN CHILDREN OF EAST CDTI
ADULT POPULATION OF CDTI SITES ONLY
INTERVIEW FOR TREATMENT
THICK BLOOD FILM
NUMBER OF TREATMENT ROUNDS
PREVALENCE AND INTENSITY
CORRELATION
ADULT POPULATION OF ALL SITES BEFORE CDTI
ADULT AND CHILDREN POPULATION OF ALL SITES AT EVALUATION
RECRUITMENT
RECRUITMENT
THICK BLOOD FILM
THICK BLOOD FILM
PREVALENCE OF ADULTS WITH HIGH AND VERY HIGH MF LOAD BEFORE CDTI IN ALL SITES
VS
PREVALENCE OF ADULTS WITH HIGH AND VERY HIGH MF LOAD AT EVALUATION IN ALL SITES
PREV. OF CHILDREN WITH HIGH AND VERY HIGH MF LOAD IN EAST NON-CDTI
VS
PREV. OF CHILDREN WITH HIGH AND VERY HIGH MF LOAD IN EAST CDTI
ADULT POPULATION OF CDTI SITES
GENERAL POPULATION OF CDTI SITES
INTERVIEW FOR TREATMENT
INTERVIEW FOR TREATMENT
THICK BLOOD FILM
THICK BLOOD FILM
COMPLIANCE TO IVM AND RISK REDUCTION ASSESSMENTS
COMPLIANCE TO IVM AND RISK REDUCTION ASSESSMENTS

LEGEND

I- Block 1 describes the first part of the study which involved recruiting adults of both sexes before and after several rounds of CDTI. These adults were screened for *L. loa* and the prevalence and intensity of infection before and after CDTI compared.

II- Block 2 correlates the prevalence and intensity of L. loa mf to oral declaration of Ivermectin treatment history

III- Block 3 compares the potential risk reduction before and after CDTI

IV- Block 4 describes the association between adherence to Ivermectin and the risk of having a defined mf load
