# Supplementary material for: Impact of repeated annual community directed treatment with ivermectin on loiasis parasitological indicators in Cameroon: Implications for onchocerciasis and lymphatic filariasis elimination in areas co-endemic with Loa loa in Africa
Source: PLoS Negl Trop Dis. 2018 Sep 18;12(9):e0006750. doi: 10.1371/journal.pntd.0006750 (PMC6161907; doi:10.1371/journal.pntd.0006750)
Supplement: S1 Table — (PDF) [file pntd.0006750.s002.pdf]

Region				Microfilaria loads				Total
				0mf/ml	1-8000 mf/ml	8001 – 30000 mf/ml	>30000 mf/ml	
North-West	Community	JATOR	Positive	100	7	2	2	111
			Percentage	90,1%	6,3%	3,6%	1,8%	100,0%
		MBIRIPKWA	Positive	41	25	2	2	70
			Percentage	58,6%	35,7%	5,7%	2,9%	100,0%
		NGOMKOW	Positive	59	7	0	0	66
			Percentage	89,4%	10,6%	0,0%	0,0%	100,0%
		NGU	Positive	63	36	8	5	112
			Percentage	56,2%	32,1%	11,6%	4,5%	100,0%
		NGURI	Positive	45	29	7	10	91
			Percentage	49,5%	31,9%	18,7%	11,0%	100,0%
		NKING	Positive	45	38	5	8	96
			Percentage	46,9%	39,6%	13,5%	8,3%	100,0%
		NTEM	Positive	64	24	3	4	95
			Percentage	67,4%	25,3%	7,4%	4,2%	100,0%
		NWANTI	Positive	59	38	9	1	107
			Percentage	55,1%	35,5%	9,3%	0,9%	100,0%
		NWAT	Positive	34	13	5	0	52
			Percentage	65,4%	25,0%	9,6%	0,0%	100,0%
		SABONGARI	Positive	204	18	4	2	228
			Percentage	89,5%	7,9%	2,6%	0,9%	100,0%
	Total		Positive	714	235	79	34	1028
			Percentage	69,5%	22,9%	7,7%	3,3%	100
South-West	Community	AJAMAN	Positive	83	5	0	0	88
			Percentage	94,3%	5,7%	0,0%	0,0%	100,0%
		AKWA	Positive	63	2	1	0	66

		Percentage	95,5%	3,0%	1,5%	0,0%	100,0%
	ARARU	Positive	26	4	0	0	30
		Percentage	86,7%	13,3%	0,0%	0,0%	100,0%
	ASSAM	Positive	53	3	1	1	57
		Percentage	93,0%	5,3%	1,8%	1,8%	100,0%
	BABI	Positive	22	4	0	0	26
		Percentage	84,6%	15,4%	0,0%	0,0%	100,0%
	BABONG	Positive	161	17	3	0	181
		Percentage	89,0%	9,4%	1,7%	0,0%	100,0%
	BACHE	Positive	120	7	1	0	128
		Percentage	93,8%	5,5%	0,8%	0,0%	100,0%
	EKONEMAN	Positive	23	1	0	0	24
		Percentage	95,8%	4,2%	0,0%	0,0%	100,0%
	KESHAM	Positive	159	12	3	0	174
		Percentage	91,4%	6,9%	1,7%	0,0%	100,0%
	MBOFONG	Positive	36	2	1	0	39
		Percentage	92,3%	5,1%	2,6%	0,0%	100,0%
	NKOGHO	Positive	89	15	3	2	107
		Percentage	83,2%	14,0%	2,8%	1,9%	100,0%
	OBONYI 1	Positive	114	6	1	0	121
		Percentage	94,2%	5,0%	0,8%	0,0%	100,0%
	OBONYI 3	Positive	149	9	1	0	159
		Percentage	93,7%	5,7%	0,6%	0,0%	100,0%
	OGURANG	Positive	75	4	0	0	79
		Percentage	94,9%	5,1%	0,0%	0,0%	100,0%
	OKPAMBE	Positive	44	1	0	0	45
		Percentage	97,8%	2,2%	0,0%	0,0%	100,0%
	TAKAMANDA	Positive	123	11	0	0	134
		Percentage	91,8%	8,2%	0,0%	0,0%	100,0%
Total		Positive	1340	103	15	3	1458
		Percentage	91,9%	7,1%	1,0%	0,2%	100
