# Supplementary material for: Impact of repeated annual community directed treatment with ivermectin on loiasis parasitological indicators in Cameroon: Implications for onchocerciasis and lymphatic filariasis elimination in areas co-endemic with Loa loa in Africa
Source: PLoS Negl Trop Dis. 2018 Sep 18;12(9):e0006750. doi: 10.1371/journal.pntd.0006750 (PMC6161907; doi:10.1371/journal.pntd.0006750)
Supplement: S2 Table — (PDF) [file pntd.0006750.s003.pdf]

Project_Region				Microfilaria loads				Total
				0 mf/ml	1-8000mf/ml	8001-30000mf/ml	>30000mf/ml	
EAST (8 years CDTI)	Community	AVIATION	Positive	67	7	1	1	76
			Percentage	88,2%	9,2%	1,3%	1,3%	100,0%
		BISSOUA 2	Positive	89	21	4	0	114
			Percentage	78,1%	18,4%	3,5%	0,0%	100,0%
		BOMBI	Positive	78	12	1	1	92
			Percentage	84,8%	13,0%	1,1%	1,1%	100,0%
		DENG-DENG	Positive	66	9	1	0	76
			Percentage	86,8%	11,8%	1,3%	0,0%	100,0%
		DOUME VILLAGE	Positive	29	1	0	0	30
			Percentage	96,7%	3,3%	0,0%	0,0%	100,0%
		KANDA	Positive	79	15	1	2	97
			Percentage	81,4%	15,5%	1,0%	2,1%	100,0%
		KOUM	Positive	37	4	0	0	41
			Percentage	90,2%	9,8%	0,0%	0,0%	100,0%
		LABBA	Positive	31	2	0	0	33
			Percentage	93,9%	6,1%	0,0%	0,0%	100,0%
		MAYOS	Positive	55	6	0	0	61
			Percentage	90,2%	9,8%	0,0%	0,0%	100,0%
		MBETHEN 2	Positive	72	17	1	1	91
			Percentage	79,1%	18,7%	1,1%	1,1%	100,0%
		MEBA	Positive	33	12	0	0	45
			Percentage	73,3%	26,7%	0,0%	0,0%	100,0%
			Positive	60	9	0	0	69

		MESSAMENA VILLAGE	Percentage	87,0%	13,0%	0,0%	0,0%	100,0%
		NDEMBA 1	Positive	78	12	0	2	92
			Percentage	84,8%	13,0%	0,0%	2,2%	100,0%
		NKOMZUH	Positive	32	11	0	0	43
			Percentage	74,4%	25,6%	0,0%	0,0%	100,0%
		NTOLLOCK	Positive	36	11	0	0	47
			Percentage	76,6%	23,4%	0,0%	0,0%	100,0%
		SOLEYE	Positive	107	11	5	0	123
			Percentage	87,0%	8,9%	4,1%	0,0%	100,0%
	Total		Positive	949	160	14	7	1130
			Percentage	84,0%	14,2%	1,2%	0,6%	100,0%
NORTH-WEST (9 years CDTI)	Community	JATOR	Positive	81	10	2	0	93
			Percentage	87,1%	10,8%	2,2%	0,0%	100,0%
		MBIRIPKWA	Positive	81	25	0	3	109
			Percentage	74,3%	22,9%	0,0%	2,8%	100,0%
		NGOMKOW	Positive	137	3	1	0	141
			Percentage	97,2%	2,1%	0,7%	0,0%	100,0%
		NGU	Positive	88	18	3	0	109
			Percentage	80,7%	16,5%	2,8%	0,0%	100,0%
		NGURI	Positive	84	23	4	1	112
			Percentage	75,0%	20,5%	3,6%	0,9%	100,0%
		NKING	Positive	56	20	7	3	86
			Percentage	65,1%	23,3%	8,1%	3,5%	100,0%
		NTEM	Positive	107	22	3	0	132
			Percentage	81,1%	16,7%	2,3%	0,0%	100,0%
		NWANTI	Positive	93	24	2	1	120
			Percentage	77,5%	20,0%	1,7%	0,8%	100,0%

		NWAT	Positive	88	8	2	1	99
			Percentage	88,9%	8,1%	2,0%	1,0%	100,0%
		SABONGARI	Positive	79	8	1	0	88
			Percentage	89,8%	9,1%	1,1%	0,0%	100,0%
	Total		Positive	894	161	25	9	1089
			Percentage	82,1%	14,8%	2,3%	0,8%	100,0%
SOUTH-WEST 2 (14 years CDTI)	Community	ASSAM	Positive	41	2	0	0	43
			Percentage	95,3%	4,7%	0,0%	0,0%	100,0%
		AYUKABA	Positive	91	5	0	0	96
			Percentage	94,8%	5,2%	0,0%	0,0%	100,0%
		BACHE	Positive	71	6	0	0	77
			Percentage	92,2%	7,8%	0,0%	0,0%	100,0%
		EBAM	Positive	70	4	0	0	74
			Percentage	94,6%	5,4%	0,0%	0,0%	100,0%
		EYANCHANG	Positive	87	2	0	0	89
			Percentage	97,8%	2,2%	0,0%	0,0%	100,0%
		KESHAM	Positive	89	5	1	0	95
			Percentage	93,7%	5,3%	1,1%	0,0%	100,0%
		MBAKEM	Positive	37	1	0	0	38
			Percentage	97,4%	2,6%	0,0%	0,0%	100,0%
		MBATOP	Positive	79	8	2	1	90
			Percentage	87,8%	8,9%	2,2%	1,1%	100,0%
		OKPAMBE	Positive	56	11	2	0	69
			Percentage	81,2%	15,9%	2,9%	0,0%	100,0%
		TABOH	Positive	55	7	0	0	62
			Percentage	88,7%	11,3%	0,0%	0,0%	100,0%
	Total		Positive	676	51	5	1	733
			Percentage	92,2%	7,0%	0,7%	0,1%	100,0%
