# Supplementary material for: Impact of repeated annual community directed treatment with ivermectin on loiasis parasitological indicators in Cameroon: Implications for onchocerciasis and lymphatic filariasis elimination in areas co-endemic with Loa loa in Africa
Source: PLoS Negl Trop Dis. 2018 Sep 18;12(9):e0006750. doi: 10.1371/journal.pntd.0006750 (PMC6161907; doi:10.1371/journal.pntd.0006750)
Supplement: S3 Table — (PDF) [file pntd.0006750.s004.pdf]

Site	Time of Screening	Non neurological Risk (%)	No risk	Total screened	Risk Difference (%)	% Potential Risk avoided amongst CDTI adherents**	Relative Risk	% Potential Prevented cases in the population***
East	Baseline Non CDTI	46 (5,1)	854	900				
	Follow up (8 years CDTI)	14 (1,2)	1121	1135	32 (3,9)	76	0,24	64
Northwest	Before CDTI	45 (4,4)	983	1028	20 (2,5)	57	0,43	39
	Follow up	25 (1,9)	1299	1324				
Southwest	Before CDTI	12 (0,8)	1446	1458	7 (0,3)	38	0,62	30
	Follow up	5 (0,5)	924	929				

*These are individuals with *L. loa* mf loads between 8000mf/ml – 30000 mf/ml
** Calculated as (1-RR)*100, where RR = relative risk
*** Calculated as P*(1-RR)*100, where P = IVM compliance, RR = relative risk
