# Supplementary material for: Impact of repeated annual community directed treatment with ivermectin on loiasis parasitological indicators in Cameroon: Implications for onchocerciasis and lymphatic filariasis elimination in areas co-endemic with Loa loa in Africa
Source: PLoS Negl Trop Dis. 2018 Sep 18;12(9):e0006750. doi: 10.1371/journal.pntd.0006750 (PMC6161907; doi:10.1371/journal.pntd.0006750)
Supplement: S4 Table — (PDF) [file pntd.0006750.s005.pdf]

Site	Time of Screening	Risk >30000mf/ml (%)	No Risk	Total screened	Risk Difference	% Risk avoided amongst CDTI adherents**	Relative Risk	% Prevented Risk in the population***
East	Non CDTI (baseline)	8 (0.9)	892	900	1 (0.3)	0.33	70	0.26
	Follow up	7 (0.6)	1128	1135				
Northwest	Before CDTI	34 (1.0)	3038	3072	25 (0.3)	0.3	70	0.20
	Follow up	9 (0.7)	1315	1324				
Southwest	Before CDTI	3 (0.2)	1455	1458	2 (0.1)	0.5	50	0.39
	Follow up	1 (0.1)	928	929				

*These are individuals with *L. loa* mf loads greater than 30000 mf/ml

** Calculated as (1-RR)*100, where RR = relative risk

*** Calculated as P*(1-RR)*100, where P = IVM compliance, RR = relative risk
